# The Eyes

**Published:** 1890-07

**Authors:** 


### THE EYES.

A change is occurring in the refractive media of all eyes, so that every one who attains to a ripe old age will, at some time during his or her existence, be a fit subject for the oculist—or, in other words, will need to wear glasses. In young people, this change is usually gradual and unperceived, but from middle life onward its effects are plainly apparent. Those who have normal vision while young, will require glasses for reading when they have passed beyond the age of forty, and those who are near-sighted, will need glasses in early life, if the degree of nearsightedness (myopia) be considerable, and yet they may be able to read perfectly well without glasses at fifty, or even sixty years of age. Persons who are included in this category are apt to consider themselves as lucky exceptions to general laws, and are usually very proud of their sharp sight.

But not only does the eye undergo certain normal changes as age advances, but it may be abnormally formed ; hence, optical defects are quite common in infants. The eye is a camera, and, while it may be perfectly sound, the vision may be bad because the rays of light are not focused upon the retina. Hence comes the necessity for wearing glasses, for, by placing suitable lenses before these eyes, normal, distinct vision may, within certain limits, be obtained. It is not generally known that it is the exception, and not the rule, to find eyes that are perfect in shape, or, technically speaking, that are "emmetropic." Still it does not follow that all eyes that are not perfect in shape should have glasses fitted to them, for some errors of refraction do not interfere seriously with vision, and never give rise to disease or decided discomfort to the patient; but, as a rule, persons whose eyes are "weak," or who suffer from complaints similar to those which we shall soon consider, should present themselves to some competent oculist for the detection and subsequent correction of any existing errors of refraction.

There still exists quite a prejudice in the minds of many against the use of glasses, but why such prejudice should exist is very difficult of explanation on any other grounds than willfulness and ignorance. All ophthalmologists teach the great necessity of correcting errors of refraction by wearing proper glasses, and we shall herein endeavor to show some of the undesirable, and even portentous results of permitting optical defects to go uncorrected. As a rule, glasses add nothing to the appearance of the wearer, and they are often a source of inconvenience, and, unless there is a definite object to be attained by their use, one is better without them ; but where they are indicated and advised by one competent to decide, neither vanity nor prejudice should prevent their being employed.

In general, it may be said that all errors of refraction which reduce the patient's vision to any extent below the normal, or which produce any marked change in either the near or the far points, require correction by the use of suitable glasses.

The effort of accommodation is a muscular exertion, and hence a tax upon the nervous system, and, if long continued, results in more or less exhaustion. When far-sighted eyes are used for reading or near work, for any considerable period of time, a larger flow of blood is sent to the eyes, hence, there is an increased secretion of mucus, or "watering of the eyes ;" and, if the work be still continued, dizziness, headache, a feeling of sickness, or even actual vomiting, may be induced. As excessive effort of accommodation is always associated with increased convergence, and, as a far-sighted eye must always increase its accommodation in order to gain clear vision, it naturally squints inward, and nervous twitchings of the eyelids and other portions of the face are sometimes occasioned by it.

Short sight is often hereditary or congenital, but may be acquired from prolonged straining of the eye. This condition is not infrequently the precursor of serious, and sometimes irremediable impairment of vision, and hence skilled advice and proper glasses are of highest importance to the patient in preventing the accidents to which every myopic eye is liable. There is an excessive demand made upon the muscles that converge the eyes, in the efforts made to keep them both fixed upon small objects held close to the face, and sometimes, being unable to withstand this strain, they give out, and one eye is then turned outward by the opposing muscle, forming a divergent squint. The vision should be rendered normal—except in very high degrees—by the use of concave spherical glasses, and every thing which tends to congest the eyes—such as reading or writing in the recumbent or stooping posture, or by faulty light—is to be most carefully avoided.

The far-sight of old age, is caused by a lack of power of accommodation, and, although distant vision remains unimpaired, there is a constant recession of the near point. This is first noticed when one finds that he is obliged to hold his paper farther away from his eyes than before, and that the print is not so clear as formerly. This is easily corrected by convex glasses for reading, and they should be employed as soon as the affection becomes manifest. It does not usually cause inconvenience until after the age of forty.

In astigmatism, or irregular sight, the refraction differs in different portions, or meridians, of the eye, and the retinal image is thus confused. This condition is usually congenital, and may be hereditary ; it is, however, sometimes acquired, often occurring after inflammations of the cornea, and may even be occasioned by the use of improper glasses. It is a very common optical defect, and is corrected either by cylindrical lenses, or by combining cylindrical with either spherical or cylindrical lenses.

A different refractive condition in the two eyes of the same person is quite common. One eye may be correct, and the other long-sighted or short-sighted ; or one eye may be long-sighted and the other short-sighted. Both eyes must be tested separately, and fitted accordingly.

Weakness of some one or more of the ocular muscles, is very often a complication of some error of refraction. In this condition there is a continual strain upon the weaker muscle in order to do its work, and this alone will cause very many headaches, neuralgias, and general nervous symptoms. We have already considered this subject in cases where the irregular action of the muscles of the eyeball is sufficiently marked to produce squint, but ofttimes there is merely a loss of function which can be determined only by careful examination.

Any defect or impairment of vision, other than the farsightedness consequent upon advancing years, as soon as discovered, should be submitted to the examination and treatment of a competent oculist. Neglect in this regard is likely to work serious injury upon the afflicted.

Children should early be taught the necessity of certain simple rules touching the use of their eyes, and parents should carefully note that their requirements are heeded. It is better to have no artificial light in sleeping rooms ; but as such light is often a necessity, it should be so shielded as not to fall directly upon the eyes of the sleeper. Neither should sunlight be allowed to shine through a window upon the bed, either directly or by reflection. Where it is a necessity to sleep during the daylight hours, as is so often the case in the multifold diversities of labor in a city, the room should not be made dark. Closing the shutters and drawing the shades so as to shut out direct light will usually be sufficient, and on waking, the change to the strong midday light will be less trying to the eyes.

In a general way, it may be said, that whatever pains the healthful eye should be avoided. This includes the reading of very fine or poor print ; especially when the attempt is made on a railroad train or other conveyance, where the vibration of the vehicle constantly changes the focus, and makes it difficult to follow the lines, as well as reading at twilight, or by any other imperfect illumination.

In reading or writing, the light should come obliquely from the side, and fall upon the surface of the paper so as to fully illumine it, with the reflection passing away at an angle without striking the reader in the face. The reflection from white paper is injurious. The sight should never be taxed during general weakness, or in convalescence, as the nerves and muscles share the general debility, and are easily overtaxed, nor is it advisable to read while lying down, or in a stooping posture. One of the advantages of the type-writer is, that it allows an erect position.

Many eyes are seriously strained and injured by deferring the use of glasses after the focus has changes by purely natural causes. This is hurtful, as their function is to assist and save the eyes. If properly treated these organs will remain efficient till life's close. The period when spectacles become a necessity varies much, but with normal and well matched eyes, it may be expected about the age of forty-five. Sometimes it will come later or even sooner.

The selection of proper lenses at this time is not a difficult matter. Those of low power should be used at first, since the purpose is not to magnify objects, but to render them clear and distinct. See that the print you read is clear, and test the glasses by wearing them for at least half an hour, and under a variety of conditions. If they bring a sense of relief to the eye while reading, and can then be laid aside without derangement of vision, they are right.

